# Relationships of accelerometer-based measured objective physical activity and sedentary behaviour with cognitive function: a comparative cross-sectional study of China’s elderly population

**DOI:** 10.1186/s12877-020-01521-y

**Published:** 2020-04-22

**Authors:** Zhi-jian Wu, Zhu-ying Wang, Bing-qian Hu, Xu-hui Zhang, Fan Zhang, Hou-lei Wang, Fang-hui Li

**Affiliations:** 1 School of Sport Sciences, Nanjing Normal University, No. 1 Wenyuan Road Qixia District, Nanjing, 210046 China; 2 Dongguan Primary School, Zhengzhou, China; 3 School of Police Skills and Tactics, Nanjing Forest Police College, Nanjing, China; 4 Physical Education Department, Nanjing University of Posts and Telecommunications, Nanjing, China

**Keywords:** Accelerometer, Elderly population, Physical activity, Cognitive ability

## Abstract

**Background:**

This study explored the effects of physical activity and sedentary behaviour on the decline of cognitive ability among the elderly. To compensate for the limitations of self-reported physical activity, objective measures were used.

**Methods:**

A cross-sectional survey of 308 aged people mean 68.66 ± 5.377 years, in Nanjing, China, was conducted. Physical activity was measured using the ActiGraph GT3X+, and cognitive function was measured using the Montreal Cognitive Assessment.

**Results:**

The overall participant model, adjusted for age, BMI, education, and monthly average income, found that light physical activity (β = 0.006, *p* < 0.01), moderate-vigorous physical activity (β = 0.068, *p* < 0.001), and total physical activity (β = 0.006, *p* < 0.01) had a significant linear relationship with cognitive ability, while sedentary time did not (β = − 0.020, *p*>0.05). Further, light physical activity only affects the cognitive ability of elderly females (β = 0.006, *p* < 0.05). There was an inverted ‘U’ association between moderate-vigorous physical activity and cognitive ability. The association models found that moderate-vigorous physical activity in the 22.13 min·day^− 1^~38.79 min·day^− 1^ range affected cognitive ability most beneficially, with the highest beta coefficient among all groups (β = 0.091, *p* < 0.05).

**Conclusions:**

While physical activity can significantly improve cognitive ability among the elderly, sedentary behaviour is associated with decreased cognitive function across genders.

## Background

While an increasing aging population is a current global problem, it is particularly serious in China [1]. Age-related cognitive decline is associated with the loss of independence, the loss of quality of life, premature death, and higher healthcare costs [2]. The cognitive decline of the elderly is 0.04–0.05 standard deviations per year, and more pronounced in patients with mild cognitive impairment, leading, in turn, to dementia [3]. It is estimated that the population of the elderly in China will reach 402 million in 2040, and the number of dementia cases will increase significantly, becoming one of the biggest health threats in the nation [4]. Therefore, reducing the burden of cognitive impairment and its sequelae in a rapidly aging population should be a priority for public health policy makers.

As there is no known treatment for dementia yet, greater attention has recently been given to its prevention. A crucial initial step to prevent a disease is to identify its risk factors and implement interventions. One of the modifiable risk factors for dementia is physical inactivity, a phenomenon that is regrettably common worldwide [5]. Sedentary behaviour was reportedly associated with worse cognitive function [6]. A meta-analysis of longitudinal studies by Blondell et al. (2014) found that higher baseline physical activity was associated with a 14% reduction in future dementia risk [7]. At least two systematic reviews state that physical activity is one of the seven modifiable risk factors for cognitive impairment in the elderly [5, 8]. Evidence-based studies show that physical activity and sedentary behaviour play an important role in cognitive function in older adults [9]. However, some studies have reported associations between physical activity and cognition cross-sectionally [10, 11] and longitudinally [12]. The results, however, are controversial, especially considering that most of them are observational and rely on participants’ self-reported physical activity assessments, which may be affected by recall bias, cognitive ability, health status, and other factors, especially among older people. This is a limiting factor because the current state of cognitive function may affect an individual’s ability to accurately report their activity. Therefore, some scholars believe that the effectiveness of physical activity as a protective measure to prevent cognitive decline in the elderly remains questionable [13]. Thus, to overcome the limitations of self-reported assessments, objective physical activity measurements are increasingly being used to estimate physical activity and sedentary time more accurately. Accelerometers are a useful tool in this regard, as they allow researchers to measure total physical activity (TPA), including light physical activity (LPA), moderate-vigorous physical activity (MVPA), and sedentary behaviour (SED) which are difficult to recall on self-report questionnaires [14]. Further, the difference in the impact of physical activity on cognitive function between older men and women is unclear.

In view of the above, this study of the elderly in China used a three-dimensional accelerometer to measure the amount of physical activity, then assessed cognitive ability via the Montreal Cognitive Scale (MoCA), and finally explored the relationship between physical activity and cognitive ability. It aims to deepen our understanding of the relation between good physical health and cognitive health in the context of aging. Further research is needed to understand the relationship between MVPA and its cognitive health benefits among the elderly. The results of this study can thus be used to update exercise recommendations and exercise programs for older people with cognitive impairment.

## Methods

### Sample

This study was conducted in Nanjing, a national central city in eastern Jiangsu Province in China. In 2017, the population of Nanjing reached 8.335 million residents, of whom slightly more than one-fifth (20.850%) were over 60 years of age. The study was a cross-sectional survey. A random sampling method was used to extract 400 subjects (range age 60–80, male 200) from Nanjing. The study was approved by the Human Body Committee of Nanjing Normal University and all participants provided written informed consent prior to inclusion in the study.

The inclusion criteria for participants were as follows: a) were over 60 years of age at the time of the survey; b) had been residing in the area for at least 6 months; and c) were able to communicate normally, so that their vision, hearing, and mental state allowed them to complete the cognitive test for older people. Exclusion criteria were, a) severe speech, hearing, and visual impairment; and b) a diagnosis of Alzheimer’s disease or mental illness such as severe depression or schizophrenia. The subjects’ family members were informed, and they provided consent as well. If a subject felt uncomfortable during the study, they could withdraw unconditionally at any time. In order to ensure the uniformity and representativeness of the sample distribution, the subjects were randomly selected from among the elderly aged 60–65 years, 66–70 years, 71–75 years, and 76–80 years, half of whom were male and the other half female. In all, 350 elderly people completed the survey; 42 questionnaires were eliminated as invalid, effectively retrieving 308 questionnaires, with an effective rate of 88.0% [15].

### Measurement of physical activity

We used ActiGraph GT3X + to collect physical activity estimates, which involved assessing subjects’ walking, running, and daily activities by measuring vertical acceleration or activity counts. Participants wore accelerometers for 7 consecutive days during all waking hours, except when showering or swimming. After the experiment, they were instructed to return the device immediately [16]. We defined SED as 0–100 counts per minute, LPA as 100–1951 counts per minute, and MVPA> 1952 per minute [17]. We defined non-wearing time as 0 activity counts of at least 60 min. In order for the participants’ accelerometer data to be considered valid for the analysis, subjects were required to wear the accelerometer for at least 3 days and at least 8 h per day [18]. We verified the wear time using an algorithm [19] developed by ActiLife 6 version 5.5 and Troiano and colleagues. The accelerometer’s physical activity variables were reported every 10 min as well as a daily mean. In a 10-min round, we allowed 1 or 2 min of rest below the threshold. Objective physical activity variables included mean daily minutes of unbouted (a) SED, (b) LPA, (c) MVPA, and (d) TPA.

### Measurement of cognitive function

Cognitive function was assessed using the MoCA (available at www.mocatest.org), a one-page 30-point test administered in 10 min to screen for Mild Cognitive Impairment. It achieves this by assessing the subjects for short-term memory recall, visuospatial abilities, multiple aspects of executive functions, attention, concentration, working memory, language, and orientation to time and place. Each of these domains is evaluated for a certain number of points, with higher points indicating better cognition [20].

### Statistical analysis

We conducted all analyses using SPSS Version 22.0. Multiple regression models were used to analyse the relationship between objective physical activity (SED, LPA, MVPA, and TPA) and cognitive function of the elderly.

First, the demographic variables were described and statistically analysed. Data conforming to the normal distribution were expressed by the mean ± standard deviation (Mean ± SD), and the data that did not conform to the normal distribution were described by the interquartile range. Comparing the male and female groups, the normal distribution data were analysed by a t-test, and the non-normal distribution data by the Mann-Whitney test. Second, Spearman’s correlation was used to analyse the correlation between physical activity and cognitive function. Finally, multiple linear regression was used to explore the relationship between physical activity and cognitive function among the elderly. Model 1 is a univariate model to investigate the relationship between SED, LPA, MVPA, and TPA, and cognitive function. Model 2 analyses the effects of SED, LPA, MVPA, and TPA on cognitive function with demographic factors as covariates.

## Results

### Demographic information of the subjects

Table 1 shows the demographic characteristics of the 308 respondents. The mean age was 68.66 years (SD = 5.37 years) and just under half of the sample were male (42.5%). More than half were overweight or obese at the time of this study, only 14.3% were university graduates, and 78.9% had a monthly income of more than 2001 ¥. The duration of SED, LPA, MVPA, and TPA were 590.75 ± 116.03 min/day, 171.74 ± 56.71 min/day, 46.12 ± 31.39 min/day and 216.98 ± 62.49 min/day, respectively. The average cognitive score was 24.39 (SD 3.03). The independent sample t-test found that the MVPA time of males was significantly higher than that of females (*p* < 0.05), while there were no gender differences in year, BMI, SED time, LPA, TPA, and cognitive scores (*p* > 0.05).

**Table 1:** List of basic information of respondents (*n* = 308)

	Male (*n* = 131)	Female (*n* = 177)	Total (*n* = 308)	Sig.
Year	69.24 ± 5.20	68.23 ± 5.46	68.66 ± 5.37	0.100
BMI (kg/m2)	25.38 ± 2.87	26.72 ± 20.07	26.15 ± 15.33	0.452
**BMI rating(%)**				
Normal	15.6	23.7	39.3	–
Overweight	18.2	23.1	41.2	–
Obesity	8.8	10.7	19.5	–
**Highest level of education (%)**				
Elementary school	3.6	8.4	12.0	–
Junior high school	14.9	23.1	38.0	–
Senior high school	13.3	22.4	35.7	–
University school	10.7	3.6	14.3	–
**Average monthly income(%)**				
Below ¥1000	5.1	5.5	10.7	–
¥1001–2000	2.3	8.1	10.4	–
¥2001 or more	35.0	43.8	78.9	–
SED (min·day^−1^)	586.01 ± 129.58	594.26 ± 105.14	590.75 ± 116.03	0.539
LPA (min·day^− 1^)	161.32 ± 47.71	179.46 ± 61.54	171.74 ± 56.71	0.172
MVPA (min·day^− 1^)	50.00 ± 31.07	43.25 ± 31.41	46.12 ± 31.39	0.040
TPA (min·day^− 1^)	211.32 ± 59.91	221.17 ± 49.13	216.98 ± 62.49	0.080
Cognitive ability	24.69 ± 2.74	24.18 ± 3.22	24.39 ± 3.03	0.133

Note: *SED* sedentary behavior, *LPA* light physical activity, *MVPA* moderate-vigorous physical activity, *TPA* total physical activity

### Physical activity and cognitive ability

This study used a linear regression model to analyse the relationship between physical activity and cognitive ability in the elderly. As can be seen from Table 2, for the overall participants, Model 1, with F = 183.381, *p* < 0.001, can explain 64.4% of the cognitive variability of the elderly; after correction, it is still found that Model 2 can explain 69.4% of the cognitive ability of the elderly. After controlling for confounding factors, LPA (β = 0.006, *p* = 0.003), MVPA (β = 0.065, *p* = 0.000), and TPA (β = 0.006, *p* = 0.002) had a significant association with cognitive ability while SED did not. When the other factors were kept constant, the cognitive performance of the elderly increased by 0.06 points, 0.65 points, and 0.06 points, respectively, for each 10 min/d increase in LPA, MVPA, and TPA. Although LPA and TPA can increase the cognitive scores, the regression coefficient is poor; MVPA time is better for improving cognitive ability. Therefore, it is recommended that the elderly regularly engage in MVPA to delay the decline in cognitive ability.

**Table 2:** Results of linear regression analysis of the impact of physical activity on cognitive ability

	model		Β	SE	Sig.	*R*^2^
Total	1					0.644
		SED	−0.020	0.001	0.061	
		LPA	0.006	0.003	0.003	
		MVPA	0.068	0.004	0.000	
		TPA	0.007	0.002	0.002	
	2					0.694
		SED	−0.020	0.010	0.065	
		LPA	0.006	0.002	0.003	
		MVPA	0.065	0.004	0.000	
		TPA	0.006	0.002	0.002	
Male	1					0.545
		SED	−0.003	0.001	0.029	
		LPA	0.006	0.002	0.116	
		MVPA	0.053	0.007	0.000	
		TPA	0.008	0.004	0.036	
	2					0.615
		SED	−0.002	0.001	0.121	
		LPA	0.004	0.003	0.184	
		MVPA	0.053	0.005	0.000	
		TPA	0.008	0.004	0.034	
Female	1					0.716
		SED	0.000	0.001	0.930	
		LPA	0.006	0.003	0.025	
		MVPA	0.079	0.006	0.000	
		TPA	0.006	0.003	0.039	
	2					0.752
		SED	−0.001	0.001	0.468	
		LPA	0.006	0.003	0.029	
		MVPA	0.074	0.005	0.000	
		TPA	0.006	0.003	0.021	

Note: Model 1: Uncorrected; Model 2: Corrected age, BMI, highest education, monthly average income. *SED* sedentary behavior, *LPA* light physical activity, *MVPA* moderate-vigorous physical activity, *TPA* total physical activity

In the case of men, Model 1 found that SED (β = − 0.003, *p* = 0.029), MVPA (β = 0.053, *p* = 0.000), and TPA (β = 0.008, *p* = 0.036) had a significant association with cognitive function. After controlling for confounding factors, Model 2 can account for 61.5% of the variation in cognitive ability. While MVPA (β = 0.053, *p* = 0.000) and TPA (β = 0.008, *p* = 0.034) had a significant association with cognitive function, SED and LPA did not. In the case of women, Model 1 found that LPA (β = 0.006, *p* = 0.025), MVPA (β = 0.079, *p* = 0.000), and TPA (β = 0.006, *p* = 0.039) had a significant association with cognitive function while SED did not. These relationships remained significant after adjustment for gender, age, BMI, highest education, and monthly average income in Model 2 (LPA: β = 0.006, *p* = 0.029; MVPA: β = 0.074, *p* = 0.000; TPA: β = 0.006, *p* = 0.021). For both male and female older adults, MVPA improves cognitive performance better than LPA and TPA.

### Relationship of MVPA on cognitive ability of the elderly

In order to further refine the relationship between improved cognitive ability and MVPA, the MVPA time data of all subjects was divided into four groups according to the interquartile range (quartile 1: Q1; quartile 2: Q2; quartile 3: Q3; quartile 4: Q4); the linear regression results are shown in Table 3. Model 1 is a univariate model, Model 2 analyzes the effects of quartile of MVPA on cognitive function with demographic factors as covariates. Model 1 found that Q2 (β = 0.100, 95% CI: 0.024–0.176), Q3 (β = 0.091, 95% CI: 0.043–0.140), and Q4 (β = 0.032, 95% CI: 0.014–0.051) had a significant association with cognitive function. Adjusted linear regression models revealed remained significant associations between Q2 (β = 0.091, 95% CI: 0.009–0.172), 3 (β = 0.079, 95% CI: 0.036–0.122),4 (β = 0.031, 95% CI: 0.014–0.48) of MVPA and cognitive function of older adult when controlling for age, sex, education, BMI, monthly average income (Table 3, Model 2). The study indicated that higher MVPA quartiles were independently associated with better maintenance of cognitive function (Q 2, 3, and 4 of MVPA, *P* < 0.05) in older adults. It is also show that Q2 of MVPA improves cognitive performance better than Q1, Q3, and Q4 of MVPA. The Q4 group had the least association with improved cognitive ability in the elderly. It indicated that there was an inverted ‘U’ dose-association relationship between MVPA and cognitive ability of the elderly. Therefore, MVPA in the range 22.13~38.79 min per day was the most favourable for improving cognitive ability among the elderly.

**Table 3:** β of MVPA impact on cognitive ability (95% CI)

Model	Q1	Q2	Q3	Q4
M1	0.057(−0.038~0.152)	0.100^*^(0.024~0.176)	0.091^*^(0.043~0.140)	0.032^*^(0.014~0.051)
M2	0.089 (0.000~0.176)	0.091^*^(0.009~0.172)	0.079^*^(0.036~0.122)	0.031^*^(0.014~0.048)

Note: *: *P* < 0.05; M1: uncorrected; M2: corrected gender, age, BMI, highest education, monthly average income. Quartile 1: Q1; quartile 2: Q2; quartile 3: Q3; quartile 4: Q4. The mean quartile of MVPA time per day (min·day^−1^): Q1: MVPA < 22.13; Q2: 22.13 ≤ MVPA < 38.79; Q3: 38.79 ≤ MVPA < 65.43; Q4: MVPA ≥65.43

## Discussion

This cross-sectional study’s results show the following: First, physical activity has a positive relationship with cognitive ability, while sedentary time tends to lead to a decline in cognitive ability. Second, there are gender differences in the impact of physical activity on cognitive ability among the elderly. While MVPA and overall activity can improve the cognitive ability of elderly males, among elderly females, LPA, MVPA, and total activity can all promote it. Finally, it was found that there was an inverted ‘U’ relationship between MVPA and the cognitive ability of the elderly. MVPA of 22.13~38.79 min per day can optimally improve the cognitive ability of the elderly. Therefore, in order to delay cognitive decline among the elderly, interventions should be implemented to promote MVPA among them.

The present study arrived at the result that LPA has a significant impact on cognitive abilities in older adults. LPA preserves cognitive function in the elderly by maintaining the volume of the hippocampus and of grey matter in the frontal, parietal, and temporal cortices Existing research shows that a larger hippocampus and higher fitness levels lead to better spatial memory performance. Overall, LPA seems to affect the brain in a way that translates into preserving cognitive function [21]. The results of this study are consistent with those of Stubbs et al., involving 274 elderly people who wore accelerometers for seven consecutive days, and whose cognitive abilities were assessed using the AD8 scale [12]. The results showed that LPA is an independent factor that has a unique protective effect on cognitive ability among the elderly. Another recent study also pointed out that LPA is associated with better cognitive ability in the elderly [22]. However, Kerr and colleagues found no correlation between LPA and cognitive ability in the elderly in a cross-sectional study of 215 elderly people over 65 years of age [23]. Therefore, any inconsistencies among studies may be due to differences in cognitive assessment methods, gender differences, and age differences in subjects.

However, it is worth noting that a stratified study of males and females revealed that there is a gender difference in the influence of physical activity on the cognitive ability of the elderly. While LPA is beneficial to the cognitive ability of elderly females, it has no effect on males. In addition, the study found that MVPA improved the cognitive ability of elderly women better than men. Thus, the association between physical activity and cognitive abilities of the elderly may be regulated by gender. However, scholars at home and abroad rarely pay attention to the gender differences in the impact of physical activity on cognitive ability of the elderly. At present, the real cause of the gender difference in the impact of physical activity on cognitive ability is not known. How this effect works is beyond the scope of this study and requires further research.

Our research shows that MVPA and TPA are positively correlated with cognitive ability in the elderly. The results are consistent with those of previous studies. One study indicates that higher MVPA% leads to a 39% reduction in the risk of cognitive impairment in adults, and a 47% reduction in memory and executive function decline; MVPA can significantly reduce the risk of cognitive decline in the elderly (Relative risk [RR] = 0.85, 95% CI: 0.75–0.95) [24]. Another study showed that older adults who engaged in moderate physical activity and vigorous physical activity had lower rates of cognitive impairment and performed better in memory and executive functions than those who engaged in only LPA [11]. Thus, it may be surmised that higher MVPA can promote the maintenance of cognitive ability in the elderly, and further, MVPA improves the cognitive function of the elderly better than LPA and TPA. This may suggest that since the intensity of activity is likely to be a key factor in promoting the relationship between physical activity and cognitive ability, MVPA is more effective in maintaining cognitive ability in the elderly and should therefore be promoted among them. Although most studies have pointed out the positive effects of MVPA on cognitive abilities in the elderly, some differences have been found in Umegaki and other studies, that there is no significant correlation between MVPA and cognitive ability in the elderly [25]. These differences may be due to the choice of the study population, the measurement of physical activity and cognitive ability, and the limitations of the methodology.

Epidemiological studies have shown that there is a dose-effect relationship between physical activity and cognitive ability. Loprinzi et al. used self-reported physical activity to find that there is an inverted U-shaped relationship between physical activity and cognitive ability of the elderly [26]. It concluded that 6000~7999 MVPA MET-min-month may be the best physical activity range for improving cognitive ability. Through longitudinal studies of 6452 elderly people, Zhu et al. pointed out that there is a dose-effect relationship between objectively measured MVPA% and cognitive ability of the elderly. Higher levels of MVPA% are not only conducive to better maintaining the memory and execution function of the elderly, but can also reduce the risk of cognitive impairment among them. This study found as well that there is an inverted ‘U’ relationship between MVPA and cognitive ability in the elderly, consistent with Loprinzi’s findings using subjective measures. The non-linear relationship between physical activity and the cognitive abilities of the elderly is consistent with recent findings that assess the relationship between physical activity and cardiovascular disease biomarkers and mortality [27]. They observed that higher levels of physical activity did not result in greater survival benefits than less high levels of physical activity. Similarly, a meta-analysis by Kramer et al. suggests that long-term exercise is not as beneficial as moderate-length exercise in improving cognitive performance in older adults [28]. In the process of aging, there is also an inverted ‘U’ relationship between exercise and oxidative stress-related physiological functions and quality of life. Taken together, these findings suggest that there may be an optimal dose of physical activity to improve cognitive performance in the elderly. This study concluded that MVPA between 22.13 to 38.79 min per day was the most favourable for improving cognitive ability in the elderly. According to the WHO’s global physical activity guidelines, the elderly need at least 150 min of MVPA per week for overall health, including cognitive ability. The results of this study support this recommended amount. Therefore, according to the physical characteristics of the elderly, moderate and high-intensity physical activity should be appropriately arranged to improve cognitive ability. The physical activity of MVPA at doses of 22.13 to 38.79 min per day can be recommended to optimise the cognitive ability of the elderly. This conclusion is important for older people and health promotion professionals because it is not wise for older people to adopt the common ideology of ‘more is better’.

Physical activity may ameliorate the impact of aging on the cognitive abilities of the elderly through a variety of physiological mechanisms. First, physical activity can enhance neurological connectivity. Moreover, it has been confirmed in animal experiments that physical activity can facilitate a positive effect on cognitive ability by promoting mechanisms such as nerve conduction, synapse formation, angiogenesis, and the release of neurotrophic factors [29]. Second, physical activity can enhance cerebral cortical plasticity, that is, physical activity may help balance the detrimental effects of aging and neurodegenerative diseases on neuroplasticity and function [30]. Third, LPA or general activity is associated with lower levels of the plasma inflammatory marker c-reactive protein, a hallmark of systemic inflammation, often associated with cognitive decline. The findings suggest that physical activity may be beneficial in reversing the negative consequences of systemic inflammation [31]. Finally, studies have pointed out that gait speed is related to cognitive ability [32]. Increasing habitual activity is associated with faster gait speed. More LPA and TPA may represent more habitual activities, and individuals involved in more habitual activities may have a faster gait speed and better cognitive ability, which may suggest that LPA and TPA are the link between activity and cognitive ability.

An advantage of this study is that it is based on data obtained by using an objective measurement tool, the ActiGraph GT3X+, rather than self-reported measurements. It uses this data to explore the relatively accurate relationship between physical activity, sedentary behaviour, and cognitive ability among the elderly in China’s aging society, and to reveal the exercise-related gender differences in this population, providing better evidence to support cognitive improvement and prevention of neurodegenerative diseases.

However, there are some limitations in our design that should be noted. First, accelerometers cannot capture upper limb movements and thus may underestimate MVPA time; second, the cognitive ability assessment is of a single and cross-sectional design, therefore prospective and longitudinal experimental studies are needed in the future to further validate the causal relationship between physical activity and cognitive ability of the elderly; last, the relatively small sample size may also affect the outcome of sedentary behaviour, and future research should further expand the study area and the number of subjects in order to obtain more universal results.

Therefore, in conclusion, in order to delay cognitive decline among the elderly, policy makers should actively promote physical activities among them, especially MVPA, such as jogging, and square dancing. Even non-exercise, daily life activities such as walking, carrying loads such as while shopping, and using public transport systems, can improve the cognitive ability of the elderly. We thus recommend that public health researchers and officials should create a supportive environment for the elderly in public spaces, conducive to increased physical activity levels, and thus improved cognitive ability among this population.

## Conclusion

This study confirmed that there is positive association between physical activity and cognitive function in older adults. Further, the linear relationship between MVPA (22.13~38.79 min/day) and cognitive ability among the elderly is better than between LPA and TPA. There is an inverted ‘U’ - shaped dose relationship. Sedentary behaviour is associated with decreased cognitive function. Light physical activity is only positively correlated with cognitive function in elderly women.

## Data Availability

The datasets generated and/or analyzed during the current study are not publicly available due to maintain participant privacy and confidentiality requirements but are available from the corresponding author on reasonable request.

## Abbreviations

TPA: Total physical activity
LPA: Light physical activity
MVPA: Moderate-vigorous physical activity
SED: Sedentary behavior
MoCA: Montreal Cognitive Scale
Q: Quartile
